# Communication Support Needs in Adults with Intellectual Disabilities and Its Relation to Quality of Life

**DOI:** 10.3390/ijerph17207370

**Published:** 2020-10-09

**Authors:** Juan Carlos García, Emiliano Díez, Dominika Z. Wojcik, Mónica Santamaría

**Affiliations:** 1 Fundación Grupo AMÁS Social, 28914 Madrid, Spain; jc.garcia@grupoamas.org; 2 Institute for Community Inclusion (INICO), University of Salamanca, 37005 Salamanca, Spain; d.z.wojcik@usal.es (D.Z.W.); msantamariado@upsa.es (M.S.); 3 Faculty of Education, Universidad Pontificia de Salamanca, 37002 Salamanca, Spain

**Keywords:** intellectual disability, communication support needs, quality of life

## Abstract

Research suggests that individuals with intellectual disabilities (ID) experience difficulties in communication, ranging from intelligibility issues to more severe problems in the use and comprehension of spoken, written or sign language. Despite the negative effects that the inability to communicate have on quality of life (QoL), not much research has explored the relationship between communicative competence and QoL in the adult population with ID. The aim of this study was to describe the global communication profile of a sample of 281 adults with ID recruited from Grupo AMÁS Social Foundation, who differed in their level of communication support needs (CSN). The relationships between communicative competence and CSN with QoL were further examined. The results showed lower QoL indices for those participants characterized by their limited use of discourse and inability to exhibit certain communicative purposes, with the largest differences in the dimensions of self-determination, social inclusion, interpersonal relationships, emotional wellbeing and personal development. Overall, low levels of QoL were found for all participants, with even lower scores for the group identified as having CSN. A multiple regression model revealed that having speech/discourse competence is a powerful predictor of QoL, along with the level of disability and having the communicative competences to express likes and preferences or to establish new relationships. This clear relationship between communication and QoL is an important argument for disability support services when it comes to setting communication supports as a priority and as an important preventive step towards the protection of those at risk of exclusion.

## 1. Introduction

The growing presence and visibility of persons with intellectual disabilities (ID) in our society is a remarkable milestone on the road to full inclusion. Although the research suggests that the improvement in areas such as communication skills has a direct impact on quality of life [1,2,3], hitherto, communication support needs (CSN) in adults with ID have still not been properly addressed.

Communication support needs are very frequent in people with ID. Typically, people with CSN may need support with understanding and/or expressing themselves. For example, in a recent study, Smith et al. [4] identified communication skills in a sample of 601 adults with ID, finding that 57.9% experienced communication difficulties and, in 23.5% of cases, the difficulties were of a severe nature. This high prevalence of communication difficulties in this study was related to factors such as level of ID, low social participation, challenging behaviors or a diagnosis of Down syndrome.

The difficulties with communication that people with ID may experience are diverse, ranging from the intelligibility or fluency of speech [5], through to the understanding and comprehension of spoken, written or sign language [6], to the transmission of messages or the pragmatic use of language [7]. These three aspects, i.e., receptive, expressive and pragmatic language functioning, form an important part in a person’s communicative competence that can be assessed and measured across different situations [8]. Given that the communicative competence allows us to express desires, ideas, needs, or enables us to ask questions, etc., people who have difficulties within this area are at a direct risk of exclusion. This exclusion is understood as physical, legal, financial, and attitudinal barriers that prevent individuals from being an active participant in their community [1]. Indeed, despite the rapid advances in our understanding of the challenges that people with complex communication needs have, many of these individuals continue to experience significant challenges when it comes to inclusion into different environments, such as educational, vocational, community, healthcare, etc. [9,10]. Moreover, a growing amount of empirical work, such as the one by Snowling et al. [11], demonstrated an association between children’s language delay and an increased risk of emotional and educational problems. What is more, Clegg and Ginsborg [12], further showed that these problems continue into adulthood. Studies have also shown a heightened risk of social exclusion in adults with ID, because of a reduced amount of opportunities to establish meaningful social connections and to participate in fulfilling social activities [13,14].

The degree of impact that living with a disability can have on everyday life may be explained using the idea of quality of life (QoL). Quality of life is considered a multi-layered construct [15,16,17] which entails several distinct dimensions at both individual (micro) and environmental (macro) levels [18] that interact with each other, and reflect both objective and subjective aspects. Despite the fact that there is no universally agreed definition of QoL [2,19] and despite the existence of different definitions [20,21], there is a wide consensus that QoL provides us with a robust tool that measures individuals’ health and wellbeing and is often taken into account in clinical decision making and research [22]. There also seems to be a consensus among experts (i.e., the National Joint Committee for the Communication Needs of Persons With Severe Disabilities) that any QoL consideration must include the degree to which people can communicate effectively with members of their community, with communication being regarded as both a basic need and a human right [1].

Many studies have explored QoL in different types of conditions. An important finding from these studies suggests that individuals’ level of functioning is highly correlated with their QoL [23,24,25], whereby individuals who are high functioning also show better QoL. More importantly, for this paper, in some studies, a direct relationship has been found between aspects related to communication and QoL. For example, Biggs and Carter [26] examined the subjective health and wellbeing of 389 transition-age young people with autism or intellectual disability. They found that speech, as the primary mode of communication, along with challenging behaviors, being diagnosed with autism, and age were predictive of lower ratings of wellbeing. Similarly, Davis et al. [27] found that 11 life domains were important for the QoL of children with cerebral palsy and intellectual disability, communication being one of them.

Although communicative competence has been related to QoL in children and adolescents, the literature on adults is relatively sparse. What is more, to the best of our knowledge, little research has directly explored how QoL is related to communicative competence in adults with ID who display communication needs. There are, however, indications in the literature on cognitive communication disorders that suggest that diverse difficulties in communicative competence have an impact on different aspects of QoL [28]. For example, with regard to the inability to communicate orally, Cruice et al. [29] found that aphasic people’s functional communication ability and language functioning predicted their psychological wellbeing and social health. In fact, in a review of the topic, Hilari et al. [30] concluded that communication disability, along with other factors such emotional distress/depression, extent of aphasic impairment, the presence of other medical problems and activity level, were predictors of health-related quality of life (HRQL). Additionally, Hilari and Byng [31] found that the HRQL of individuals with severe aphasia is far more compromised than in individuals with mild aphasia. This study shows that not all people with aphasia will have the same needs and that, depending on the severity of communication and language impairment, these patients’ QoL will be differentially affected.

In the literature about people with intellectual and developmental disabilities, we can also find some studies that relate communicative competence to the family quality of life (FQoL). Schertz et al. [32], for example, looked at FQoL in children with severe neurodevelopmental disabilities and significant communication needs. The results showed that the degree of communication support offered to the family was related to the overall FQoL score and individual domains of health and family relationships. In the same vein, a research review done by Saito [3] revealed that the implementation of augmentative and alternative communication from a family perspective rather thnt from an individual point of view has a direct impact on all domains of FQoL.

Finally, there is also a widespread idea among support staff for people with ID that working on improving communicative competence can be also a good way to improve QoL. For example, when Dalton and Sweeney [33] asked 138 support staff about ways to provide help in the area of communication difficulties for people with ID in residential services, 87% of them answered that setting appropriate communication goals could improve their QoL.

Given the importance of communicative competence for QoL and the lack of literature in the area, the aim of our study is to explore the consequences that living with communication needs have in different dimensions of QoL in adults with ID. Although there are various perspectives from which to measure the QoL construct, in this paper, we focus on the quality of life model proposed by Schalock and Verdugo [34]. The model is composed of eight quality of life dimensions: self-determination, rights, emotional wellbeing, social inclusion, personal development, interpersonal relations, material wellbeing, and physical wellbeing and has proven to be useful mainly because it considers quality of life not only from the perspective of health, but also because it allows for a better understanding of how to provide the necessary support to improve QoL. Thus, it considers QoL as a dynamic and multidimensional concept, with both universal and culture-tied properties, with objective and subjective components influenced by the characteristics of the person as well as contextual factors [35]. This broad perspective outlines a set of possible areas where support can be provided, which, in turn, can have a direct impact on the QoL. In fact, in Spain, Schalock and Verdugo’s model has been shown to be a useful tool in planning support for people with ID, as well as having the capacity for encouraging institutions and organizations towards the use of QoL as the outcome measurement [36,37]. Furthermore, as stated by Navas et al. [38], there is a clear alignment between the dimensions of the Schalock and Verdugo’s QoL model and the articles of the Convention on the Rights of Persons with Disabilities [39].

In summary, in this research, we first intend to describe the global communication profile of a large sample of adults with ID that differ in their needs in relation to support for communication and, second, to explore in detail the relationships between communicative competence and communication support needs in all the QoL dimensions outlined by the Schalock and Verdugo’s model. In our study, we use the term ‘communication profile’ to reflect the communicative competence that captures the classification of communication mode and communication purpose. This preliminary diagnosis would be the first step to initiate a medium-term program to improve the support in the area of communication for users of disability support services belonging to Fundación Grupo AMÁS Social.

## 2. Method

### 2.1. Participants

The sample consisted of 281 individuals (134 women and 147 men) with complex needs. Both younger and older adults with communication needs were recruited from five live-in disability support services, four occupational disability support services, and four day disability support services, all belonging to Fundación Grupo AMÁS Social in Madrid. Most of the participants (*n* = 235) resided in disability support service establishments. To ascertain whether participants had complex and communication needs, the existing assessment files of each service user were consulted. Likewise, disability percentage, coexisting conditions (for *n* = 213), dependency levels (for *n* = 276) and intensity of support (for *n* = 217), were obtained from previous cognitive, social and health assessments carried out by professionals from each disability support service. The participants’ characteristics are depicted in Table 1.

### 2.2. Instruments

#### 2.2.1. Quality of Life—San Martín Scale

To measure the QoL, we used the field test version of the San Martín Scale [40], designed specifically for individuals who cannot participate in self-report due to profound I/DD and/or barriers to communication. It is composed of eight subscales that correspond to the eight quality of life domains by Schalock and Verdugo’s model [34], and consists of a total of 95 items. The scale is a self-administered questionnaire in which a third-party respondent who knows the service user well answers questions about their QoL. Given the communicative problems of many of the participants, completing the scale based on their opinions was not possible. Thus, all items are formulated as third person declarative statements and are randomly organized by domains. The respondents are asked to give their answers on a frequency scale with four options (never, sometimes, often, and always). The scale has shown to have an adequate reliability and validity [40] and has been used across different studies that evaluate QoL in adults with ID [41,42].

#### 2.2.2. Communication Profile Questionnaire

To explore the communication profile of participants, a custom-made checklist adapted from the Communication Device Use Checklist [43] was built with twelve yes/no questions about how the person communicates (communication modes checklist). Eleven questions were about the communication purpose and how the person communicates it (communication purpose checklist); one yes/no question asked if the person had communication support needs (a binary variable that was used in the analysis); another question checked previous participation in augmentative and alternative communication (AAC) interventions; and two final, broad questions assessed in detail the perception of professionals as to whether or not their disability support services were implementing good practice on cognitive accessibility and AAC training. The checklists and the two final questions can be consulted in the Supplementary Materials.

### 2.3. Procedure

The evaluation was carried out by a group of ninety-six professional supporting staff, working in the participating disability support services. Of these, 77.1% were women (*n* = 74) and 22.9% were men (*n* = 22) and their age ranged between 22 and 56 years (*M* = 34). As for the qualifications, almost half (*n* = 49) were direct care professionals with a degree related to social healthcare (51%): 27 were social integration technicians (28.1%), 11 were psychologists (11.45%) and 9 were managers (9.4%). The staff’s experience in providing support for people with intellectual disabilities ranged from 2 to 21 years.

The type of relationship between the healthcare workers and the person with an intellectual disability was, in all cases, professional in nature and, in all cases, the person with an intellectual disability was someone who the participating professionals knew well and they had provided support for the person for at least six months.

The evaluation was completed by the professionals within a period of 4 months, during the months from February to May 2015, using a custom-made Microsoft Excel template to record the data for each participant.

This study was approved and regulated by a collaboration agreement between the Fundación Grupo AMÁS and the first author, and all the procedures performed in this research were in accordance with the ethical standards as laid down in the 1964 Declaration of Helsinki and its later amendments or comparable ethical standards.

### 2.4. Data Analysis

Data were analyzed using R [44]. The significance level set by the researchers to conduct the statistical analyses was = 0.05.

Participant communication profiles were analyzed using chi-squared tests with the number of participants as dependent variables and the CSN group to which they belonged as the independent variable. Standardized differences were calculated according to Austin [45].

In the analysis of communication needs and QoL, the global index of QoL as well as the QoL scores of each dimension was used as dependent variables and CSN group belonging as an independent variable. For MANOVA (multivariate analysis of variance) analysis we used R package MANOVA.RM to calculate a modified ANOVA-type statistic (MATS) for multivariate designs [46] as it is applicable for non-normal error terms, different sample sizes and/or heteroscedastic variances, and *p*-values were calculated based on a parametric bootstrap approach with 10,000 iterations.

The Quality of Life Index (QLI) was used as a dependent variable in all the regression models. The selection of variables that were due to be included in the regression models was done with the glmulti function of the glmulti package. This function performs an exhaustive search for the best subsets of the variables in ‘x’ for predicting ‘y’ in linear regression. We used a branch-and-bound algorithm (the leaps function from the leaps package). Due to violations of assumptions of heteroscedasticity, normal distribution of residuals and the presence of outliers, we calculated bivariate and multiple linear regression coefficients with robust statistical methods using the R package robustbase (function lmrob), which provides different robust regression techniques (e.g., MM estimation) as well as robust univariate and multivariate methods. The function bootcoefs from the package complmrob was used to bootstrap the regression coefficients of robust linear regression models.

## 3. Results

### 3.1. Participant Communication Profile

First, we were interested in exploring the participants’ communication profiles, especially of those who were identified by the professionals as having communication support needs. It is noteworthy that direct care professionals identified as many as 182 people (64.8% of the total sample) as individuals with communication support needs (CSN group). This group included individuals who were supported, although not sufficiently, and those who did not receive support at all.

Table 2 shows the communication modes used by the participants according to their communication needs.

As can be seen in Table 2, participants in the CSN group were mostly people who did not use speech or writing and used mainly single words and gestures. Pictograms were used equally in both groups and communication devices of different complexities and emails had a rather low usage rate in both groups (<10% in all cases). Finally, there was also a notable difference in the use of the telephone in favor of the non-CSN group.

It was also interesting to explore the communicative purposes of the participants. Table 3 shows the percentage of participants in each group that exhibit a certain communicative purpose.

Here, we can observe that, in the CSN group, the percentage of participants exhibiting communication purposes was generally lower than in the non-CSN group. In a way, this is a logical result and indicates the validity of the classification made by professionals when determining whether or not a participant needs support in communication. However, for communication purposes, such as expressing needs and desires, asking for help, or showing likes and preferences, the standardized differences were low. Therefore, it could be said that people who should be supported in communication are largely successful in expressing these fundamental communication purposes. Nevertheless, for other communication purposes, such as expressing opinions, exchanging information, expressing feelings, talking to family and friends, chatting with people in their environment and being able to have new relationships, the differences between the groups were larger.

Globally, the CSN group was, therefore, characterized by a limited use of discourse and reliance on gestures and, although these limited modes of communication may serve certain basic communicative purposes, the limited communication profile could probably directly influence some dimensions of QoL such as interpersonal relationships, inclusion, rights, or emotional wellbeing.

### 3.2. Communication Needs and Quality of Life Index

Once the profile of the group with needs had been identified, it was important to explore the impact of the communication difficulties on their QoL.

The mean QLI, a standard score (with an average of 100 and a standard deviation of 15) of the total sample reached an average value of 92.1 (*SD* = 16.1; mean percentile = 36.9). Therefore, the QoL profile of the participants is considered slightly low. Indeed, as can be seen in Table 4, the scores for each of the QoL dimensions did not exceed the standard score of 10 (the standard scores on the San Martín Scale have a mean of 10 and a standard deviation of three) with the dimensions of physical wellbeing, material wellbeing and interpersonal relationships scoring the lowest (<9).

To further explore the differences in QoL as a function of CSN, a one-factor MANOVA analysis was conducted, showing that there were significant differences in QoL scores across the different dimensions (MATS = 871.2; *p* < 0.001). As presented in Table 4, post-hoc mean difference Welsch’s *t* tests showed significant differences in QLI and all QoL dimensions between groups with and without CSN. Large differences (*d* > 0.80) were found for self-determination, social inclusion, interpersonal relationships, emotional wellbeing and personal development. The QLI of the participants of the CSN group was rather low and significantly lower than that of the non-CSN group.

Additional MANOVAs showed a significant interaction of CSN with the level of disability (MATS = 45.65; *p* = 0.003) and a non-significant interaction with age (MATS = 3.09; *p* = 0.819).

### 3.3. Quality of Life Relative to Communicative Profiles

**Figure 1:**
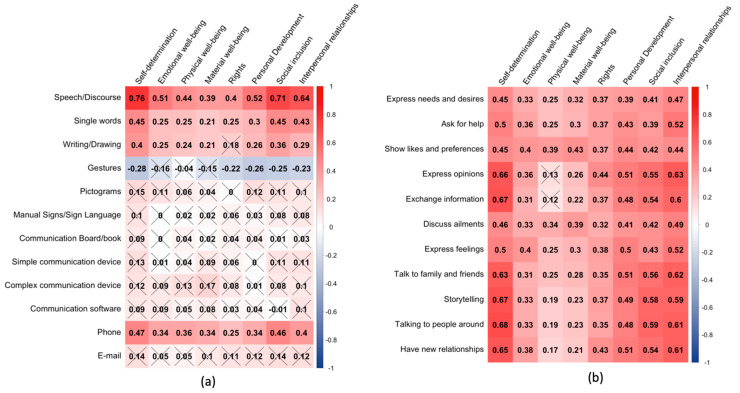
Correlograms representing point-biserial correlations among (**a**) modes of communication and (**b**) purposes of communication and quality of life dimension standard scores. The intensity and color of squares represent the magnitude and sign (red = positive and blue = negative) of the correlation, respectively. A crossed value indicates a non-significant correlation (*p* > 0.05; Bonferroni–Holm correction).

In order to further explore the relationship of QoL with the communicative profiles of the sample, point-biserial correlations were carried out between the items of the communicative profile (modes and purposes) and the standard scores in the different dimensions of quality of life. Figure 1 shows two correlograms representing the correlations of communication modes and purposes with the eight quality of life dimensions.

With regard to the modes of communication (Figure 1, panel a), it can be seen that the dimensions of self-determination, social inclusion and interpersonal relationships showed significant and greater correlations with the most complex modes of communication, with values greater than 0.64 for speech/discourse, and ranging from 0.29 to 0.47 for single words, writing/drawing and the use of a phone. The rest of the QoL dimensions showed correlations from 0.21 to 0.52 with that set of communication modes. Noteworthily, there was a moderate, though significant, negative correlation between the use of gestures and all QoL dimensions except physical and material wellbeing.

In the case of communication purposes, a very similar pattern was obtained, but with stronger correlations (Figure 1, panel b) and with almost all correlations being significant. Again, self-determination was the dimension that showed the highest correlations, followed by interpersonal relations, social inclusion and personal development dimensions.

Overall, this pattern of results points to the importance of having the ability to use some modes of communication (e.g., speech/discourse) and the need for work towards promoting certain communication purposes as a possible route towards improving QoL. This is particularly the case for the dimensions of self-determination, interpersonal relations and social inclusion that seem quite dependent on an adequate communication profile.

### 3.4. Personal and Communicative Factors as Predictors of Quality of Life

The second objective of this work was to explore how personal and communication factors impact the QoL of the participants with and without CSN. For this purpose, we carried out a multiple regression analysis with the QLI as a dependent variable and the demographic variables (age, sex, level of disability, number of disabilities, and dependency level) and communication modes (the twelve modes are displayed in Panel b of Figure 1) as independent variables.

In order to find meaningful predictors for QoL, we first tested our set of independent variables in each group by bivariate robust regressions using the function lmrob within the R package robustbase. We obtained the coefficient of determination (*R*^2^) as a measure of the explained variance by each independent variable. Table 5 shows the results of this analysis.

We then selected the predictors that had a *R*^2^ ≥ 0.04, which is the recommended minimum effect size representing a “practically” significant effect for social science data [47]. With those predictors, the best subset regression procedure [48] was used to find out the best-fit model from all possible subset models according to goodness-of-fit criteria. Specifically, both the Bayesian information criterion (BIC) and Akaike information criterion (AIC) were evaluated for models selected with a branch-and-bound algorithm and no interactions were considered, in order to build a parsimonious (simple) and a complex model, respectively. The complex model retained 10 variables, while the simple retained only five (see Table 6).

The two models were fitted with robust regression techniques, verifying the reduction in robust deviance achieved in comparison with an intercept-only model for both. Moreover, a significant reduction in robust deviance for the simple model with respect to the complex model was found (deviance-type test (5268) = 30.56; *p* < 0.001); thus, we will report the results of the most parsimonious model only.

In Table 7, bootstrapped regression coefficients are shown for the simple model fitted with the whole sample. Multiple *R*^2^ showed that the model explained 66% of the variance, denoting a large effect. All predictor variables except communication software use were significant for predicting QLI. Having competences for speech/discourse and for showing likes and preferences showed a large effect with increments of one for the standard deviation of QoL. Similarly, the use of communication to initiate new relationships showed a significant effect, although of a more moderate size. The level of disability showed a small negative effect on QoL.

In Table 8, bootstrapped regression coefficients are shown for the simple model fitted with the CSN group data. Multiple *R*^2^ showed that the model explained 54% of the variance, denoting, again, a large effect. In this case, all the predictors were significant, with similar values as the model with the total sample. The use of communication software was significant, with a large effect, although it also had a wide confidence interval.

### 3.5. Professionals’ Perceptions: ACC Use and Training

Finally, we looked at the opinions of the professionals on the situation of cognitive accessibility and support in the environment for communication and training on the AAC necessary to support people with CSN. The results are shown in Table 9.

In general, it was verified that a low percentage of people in the CSN group had received some kind of intervention by way of AAC. The supporting staff reported that the disability support services were adapted from the point of view of cognitive accessibility for a little more than half of the persons in the CSN group (51.1%) and in a significantly higher percentage (68%) in the group without CSN. Finally, with regard to the question on the training received in relation to the profile of the person, it was verified that the professionals consider the training received to be much less adequate in the case of the group of persons with CSN.

## 4. Discussion

This research aimed to describe the communication profile in adults with intellectual disabilities who differ in their degree of required communication support needs, as well as to explore the relationship between their communication profile and their quality of life.

On the one hand, it was possible to verify that the great majority of people in the CSN group (90.7%) do not use speech or discourse and depend greatly on the use of gestures to communicate. This restricted mode of communication allows them, however, to exhibit communicative purposes related to basic needs, although in a significantly lower percentage than in the group without support needs. However, there is also a notable difficulty in the ability to communicate with a more complex social function, such as expressing opinions or exchanging information, and talking with people in their surroundings or initiating new relationships.

On the other hand, a clear relationship between the communication profile and QoL has been verified, with lower QoL levels on all the dimensions of Schalock and Verdugo’s model for people in the CSN group. We also found evidence showing that the dimensions of self-determination, social inclusion, interpersonal relationships and personal development are the ones that have the strongest relationship to the communication profile. That is, the greater the ability to communicate in ID, the higher the score in the abovementioned QoL dimensions.

In general, the results are convergent with those of other studies that relate high levels of functioning to high levels of QoL, especially those studies which consider that communication skills contribute to increased self-determination [49,50,51]. With regard to this relationship, a pattern of strong positive correlations between communication purposes and self-determination have also been found, clearly showing the importance of promoting certain communication purposes that can also be considered as rights as far as the UN Convention [39] and the Communication Bill of Rights [1] are concerned. These entail rights such as the freedom and security of the person or the right to live independently and be included in the community, and communication purposes such as the right to express personal preferences and feelings, to interact socially, to maintain social closeness, to build relationships or to make comments and share opinions, to name a few.

Our results, therefore, extend the findings of previous investigations [26] into the adult population with ID, in verifying that having speech/discourse competence is a powerful predictor of QoL. Moreover, in the current work, it has been observed that the relationship between speech/discourse competence and QoL is of the greatest magnitude in the case of the dimensions of self-determination, social inclusion and interpersonal relations. In addition, other general factors related to QoL have been identified, such as having a communication profile that enables one to show likes and preferences or to establish new relationships. These findings stress, therefore, the need to intervene explicitly in the improvement of communication profiles to allow for the boosting of those QoL dimensions in ID. In the case of the CSN group, the use of AAC communication software was also a significant predictor of QoL, showing the importance of AAC systems for people with complex communication needs. Overall, our finding of a set of significant predictors has the potential to be implemented in practice, as it may aid the development of tools that target the identification of people at risk of low QoL. At the same time, the results point to different aspects of communication that might deserve special attention when it comes to the development of interventions.

Finally, the need for the training of professionals in the field of communication supports (e.g., knowledge of AAC systems) has been verified. This result is consistent with that obtained by Dalton and Sweeney [33], evidencing that support staff do not always have the training or resources to provide the support required by their users. This in turn, is likely to impinge on the staff’s possibility to adequately plan for communication support.

This study has some limitations. The first has to do with the selection of the sample. Although it is a sample with an adequate size, it was not possible to carry out totally random sampling, so there is a danger of a selection bias occurring. Along with this, the fact that the professionals carried out the assessment of both quality of life and of the identification of which group the participants belonged to (CSN/non-CSN) could limit the scope of our results. Ideally, the individuals with ID themselves should be the ones responding to the survey; however, this was not possible due to the nature of the communication difficulties of some of the participants. In this sense, we agree with Nieuwenhuijse et al. [52] in considering that research on QoL in people who cannot express themselves is a challenge and that new ways to carry out the QoL assessment should be explored. Moreover, it can be noted that our sample was very heterogeneous in terms of age, with some individuals as young as 19 and others in their seventies. Future studies could recruit participants within different age groups and directly compare whether or not there are any age effects on communication needs and their relation to QoL. Moreover, caution should be taken when generalizing our results to adults who reside in their family homes, as their reality in terms of the level of support and subsequent QoL can be different to the one experienced by individuals who are institutionalized.

## 5. Conclusions

This study has explored communication profiles and their relationship to QoL in a sample of 281 adults with ID. Overall, low levels of QoL were found for the entire sample, and especially for the group of participants who were identified as having communication support needs. More specifically, another significant finding to emerge from this study is that communicative profile is related to some of the QoL dimensions, such as self-determination, social inclusion and interpersonal relationships. This is especially important given the close link between those quality of life dimensions and some fundamental rights of people with disabilities [38,39]. Moreover, the close relationship between the communication profile and QoL in adults with intellectual disabilities, in particular for those with communication support needs, makes it clear that interventions to provide communication support for improving everyday communication should be a priority for disability support services providing services to users with these profiles. We think that a good starting point for addressing these challenges is the guidance document of the National Joint Committee for the Communication Needs of People with Severe Disabilities (NJC) [1,53], which offers information derived from a recent literature review that could be used by professionals interested in implementing effective communication services and opportunities.

This study has also found that professionals perceive the need for training on how to support people with ID as well as on how to address the necessary adaptations in the environment that facilitate communication to ensure that their right to communicate is upheld. Therefore, specific training plans are needed in relation to issues such as communication assessment, goal selection, interventions to improve communication, and interventions to improve environmental supports such as cognitive accessibility, adaptations to easy-reading or signage.

The main implication of the results of this research is to highlight, with supporting evidence, the potential for communication support interventions to improve QoL. One consequence of this research is that disability support services ought to explicitly address the communication needs of their users. In the case of AMÁS Social Foundation, the participants’ home disability support service, this research has led to the creation of group of interest in communication and cognitive accessibility, in which professionals from all the adult disability support services participate. These groups also participate in promoting measures to improve cognitive accessibility and support in the environment, as well as in providing specialized support for all the people who need support with communication. To this end, specific measures have been taken, such as promoting the participation of experts in AAC in all disability support services, and developing a training plan for AAC and accessibility for all professionals and volunteers. The next step will be to carry out a post-intervention evaluation, to assess the impact of these measures. If the relationship between communication and QoL is as strong as observed in this study, we hope to observe, in a future investigation, that increased measures to support communication will lead to improved QoL among people with complex support needs.

## Figures and Tables

**Table 1:** Participants’ characteristics.

Variable Description	Value
Mean (SD) age	42.3 (13.7)
Age range	19–71
Mean (SD) % disability	79.8 (9.7)
% disability range	37–99
Conditions associated with ID:	
Physical disability	116
Sensorial disability	70
Behavioral problems	69
Cerebral palsy	60
Dependency assessment:	
Moderate level	32
High level	112
Unknown	5
Intensity of support:	
Extensive support	133
Generalized support	84
Unknown	64

**Table 2:** Participants who use different communication modes as a percentage of each communication support needs (CSN) group.

Communication Modes	CSN			
	No (*n* = 98)	Yes (*n* = 182)	χ^2^ *p*-Value	SMD
Speech/Discourse	93.8	9.3	**	3.2
Single words	82.7	41.2	**	0.9
Writing/Drawing	37.8	7.7	**	0.8
Gestures	53.1	75.8	**	0.5
Pictograms	19.4	19.2	n.s.	0.0
Manual Signs/Sign Language	6.1	8.8	n.s.	0.1
Communication Board/book	3.1	3.9	n.s.	0.0
Simple communication device	6.1	5.0	n.s.	0.1
Complex communication device	4.1	1.7	n.s.	0.1
Communication software on a device	1.0	2.8	n.s.	0.1
Phone	61.2	14.9	**	1.1
E-mail	4.1	0.6	n.s.	0.2

n.s. = non-significant difference; ** *p* < 0.001; SMD = Standardized Mean Difference; CSN = communication support needs.

**Table 3:** Percentage of participants who are able to communicate for different purposes as a function of communication support needs.

Communicative Purposes	CSN			
	No (*n* = 98)	Yes (*n* = 182)	χ^2^ *p*-Value	SMD
Express needs and desires	99.0	80.8	<0.001	0.6
Ask for help	99.0	74.7	<0.001	0.8
Show likes and preferences	99.0	80.8	<0.001	0.6
Express opinions	93.9	46.7	<0.001	1.2
Exchange information	92.9	39.6	<0.001	1.4
Discuss ailments	98.0	70.3	<0.001	0.8
Express feelings	98.0	66.5	<0.001	0.9
Talk to family and friends	96.9	52.7	<0.001	1.2
Storytelling	90.8	25.8	<0.001	1.8
Talk to people around them	93.9	36.8	<0.001	1.5
Have new relationships	92.9	43.3	<0.001	1.3

SMD = Standardized Mean Difference (Austin, 2008); CSN = communication support needs.

**Table 4:** Mean (SD) standard quality of life scores by dimension and group.

Quality of Life Dimension	All Sample	CSN		CSN versus Non-CSN	
		No (*n* = 98)	Yes (*n* = 182)	Welsch’s *t p*-Value	Cohen’s *d* [95% CI]
Self-determination	9.4 (3.8)	12.9 (2.1)	7.6 (3.2)	<0.001	1.8 [1.5, 2.2]
Emotional wellbeing	9.2 (2.78)	10.9 (2.2)	8.3 (2.7)	<0.001	1.0 [0.7, 1.3]
Physical wellbeing	8.5 (3.4)	10.1 (2.8)	7.6 (3.4)	<0.001	0.8 [0.5, 1.0]
Material wellbeing	7.6 (3.2)	8.94 (3.0)	6.9 (3.1)	<0.001	0.7 [0.4, 0.9]
Rights	8.7 (3.3)	10.1 (2.7)	7.9 (3.4)	<0.001	0.7 [0.5, 1.0]
Personal Development	8.9 (2.9)	10.6 (2.1)	8.0 (2.8)	<0.001	1.0 [0.7, 1.3]
Social inclusion	9.1 (3.2)	11.7 (2.0)	7.7 (2.8)	<0.001	1.5 [1.3, 1.9]
Interpersonal relationships	8.2 (3.3)	10.7 (2.2)	6.9 (3.1)	<0.001	1.4 [1.0, 1.7]
Global QLI	92.1 (16.1)	104.29 (11)	85.48 (15.57)	<0.001	1.4 [1.1, 1.7]

CSN = communication support needs.

**Table 5:** Results of bivariate robust regression analyses and correlation coefficients with Quality of Life Index by group.

	Communication Support Needs			
	No		Yes	
Predictors	*R* ^2^	*r*	*R* ^2^	*r*
*Personal characteristics*				
Age	0.10 **	0.31 *	0.05 **	0.22 *
Sex	0.001	−0.005	0.01	−0.08
Level of disability	0.04	−0.06	0.12 ***	−0.32 ***
Number of additional conditions	0.04	−0.11	0.08 ***	−0.25 ***
Additional conditions				
Physical disability	0.01	−0.07	0.11 ***	−0.33 ***
Sensory—hearing	0.02	−0.15	0.01	−0.09
Sensory—visual	0.004	0.09	0.04 *	−0.18 *
Cerebral palsy	0.07	−0.25 *	0.04 *	−0.18 *
Epilepsy	0.02	−0.14	0.03 *	−0.18 *
Mental health	0.02	−0.20 *	0.02	−0.13
Down syndrome	0.05	−0.21 *	0.001	−0.01
Serious health problems	0.05	−0.20	0.01	−0.08
Behavior problems	0.11*	−0.33 ***	0.01	−0.07
*Modes of communication*				
Speech/discourse	0.33	0.24 *	0.22 ***	0.43 ***
Single words	0.08 **	−0.16	0.10 ***	0.32 ***
Writing/drawing	0.05	0.08	0.05 ***	0.22 **
Gestures	0.14 ***	−0.32 **	0.03	−0.15 *
Pictograms	0.04	−0.05	0.06 ***	0.26 **
Manual signs/sign language	0.000	0.03	0.02 *	0.14
Communication board/book	0.06	−0.20 *	0.02 *	0.15 *
Simple communication device	0.01	−0.08	0.02	0.13
Complex communication device	0.000	0.03	0.03 ***	0.15 *
Communication software on a device	0.03 ***	−0.14	0.04 ***	0.18 *
Phone	0.01	0.14	0.10 ***	0.28 ***
E-mail	0.003	0.05	0.01 ***	0.09
*Purposes of communication*				
Express needs and desires	0.23 ***	0.17	0.19 ***	0.40 ***
Ask for help	0.23 ***	0.17	0.18 ***	0.39 ***
Show likes and preferences	0.23 ***	0.17	0.27 ***	0.46 ***
Express opinions	0.000	0.14	0.15 ***	0.39 ***
Exchange information	0.01	0.15	0.11 ***	0.34 ***
Discuss ailments	0.01 ***	0.18	0.25 ***	0.42 ***
Express feelings	---	0.21 *	0.16 ***	0.39 ***
Talk to family and friends	0.02	0.13	0.17 ***	0.42 ***
Storytelling	0.01	0.11	0.09 ***	0.30 ***
Talk to people around	0.01	0.13	0.12 ***	0.36 ***
Have new relationships	0.02	0.20*	0.14 ***	0.37 ***

--- not converged; *** *p* < 0.001; ** *p* < 0.01; * *p* < 0.05.

**Table 6:** Variables included in the two best models (simple and complex) selected with best subset regression procedure.

Predictor	Simple Model (BIC)	Complex Model (AIC)
Age		YES
Level of disability	YES	YES
Physical disability		YES
Speech/discourse	YES	YES
Pictograms		YES
Communication software	YES	YES
Express needs and desires		YES
Show likes and preferences	YES	YES
Storytelling		YES
Have new relationships (yes)	YES	YES

**Table 7:** Bootstrapped robust multiple regression coefficients (whole sample).

	*B* (95 % CI)	Bias	SE (Standard Error)	*p*
(Intercept)	82.2 (69.0,96.6)	0.1	7.0	0.001 ***
Speech/discourse (yes)	17.7 (14.5,20.8)	−0.0	1.6	0.001 ***
Communication software (yes)	16.7 (−14.9,40.6)	−1.4	13.5	0.111
Show likes and preferences (yes)	15.5 (10.8,20.0)	0.1	2.4	0.002 ***
Have new relationships (yes)	4.3 (−0.02,7.7)	−0.1	1.9	0.026 *
Level of disability	−0.2 (−0.3,−0.0)	−0.0	0.1	0.009 **

*** *p* < 0.001; ** *p* < 0.01; * *p* < 0.05.

**Table 8:** Bootstrapped robust multiple regression coefficients (CSN group).

	*B* (95 % CI)	Bias	SE	*p*
(Intercept)	90.6 (71.3,114.8)	1.4	11.2	0.001 ***
Speech/discourse (yes)	18.8 (14.1,23.6)	−0.1	2.3	0.001 ***
Communication software (yes)	19.7 (−1.7,34.7)	−0.2	8.4	0.034 *
Show likes and preferences (yes)	15.0 (9.8,19.3)	−0.2	2.6	0.005 **
Have new relationships (yes)	3.8 (−0.5,7.8)	−0.2	2.1	0.039 *
Level of disability	−0.3 (−0.5,−0.1)	−0.0	0.1	0.005 **

*** *p* < 0.001; ** *p* < 0.01; * *p* < 0.05.

**Table 9:** Percentage of yes responses as a function of communication support needs.

Supports Provided	CSN			
	No (*n* = 98)	Yes (*n* = 182)	χ^2^ *p*-Value	SMD
Have the disability support services ever worked with the user using AAC (Augmentative/Alternative Communication)?	8.4	21.8	<0.01	0.38
Are the disability support services adapted from the point of view of cognitive accessibility for the user?	68.0	51.1	<0.01	0.35
Is the training received to provide communication support (e.g., AAC) adequate, taking into account the person’s profile?	35.4	14.1	<0.001	0.51

## Supplementary Materials

The following are available online at https://www.mdpi.com/1660-4601/17/20/7370/s1, Document S1: Communication profile questionnaire.
