# Supplementary material for: Communication Support Needs in Adults with Intellectual Disabilities and Its Relation to Quality of Life
Source: Int J Environ Res Public Health. 2020 Oct 9;17(20):7370. doi: 10.3390/ijerph17207370 (PMC7601275; doi:10.3390/ijerph17207370)
Supplement: Supplementary file 1 [file ijerph-17-07370-s001.pdf]

COMMUNICATION PROFILE QUESTIONNAIRE

Participant #ID: _________

Privacy Statement

Please do not write any identifying marks on the survey as participants are meant to be anonymous. All information will be kept confidential. Any concerns can be communicated to Juan Carlos García. Thank you for your time and cooperation.

Answer the following questions by placing a mark on the most appropriate answer (or use the Microsoft excel template provided)

How does the person communicate?
Choose the relevant method of communication (communication mode). Mark YES/NO.

COMMUNICATION MODES CHECKLIST	
Speech/Discourse	☐ YES ☐ NO
Single words	☐ YES ☐ NO
Writing / Drawing	☐ YES ☐ NO
Gestures	☐ YES ☐ NO
Pictograms	☐ YES ☐ NO
Manual Signs / Sign language	☐ YES ☐ NO
Communication Board / book	☐ YES ☐ NO
Simple Communication Device	☐ YES ☐ NO
Complex Communication Device	☐ YES ☐ NO
Communication software on a device	☐ YES ☐ NO
Phone	☐ YES ☐ NO
E-mail	☐ YES ☐ NO
Other (specify)	

Indicate if the person is able to communicate the following purposes (using a device or not). Also indicate communication mode that the person uses to communicate each purpose.

COMMUNICATION PURPOSE CHECKLIST		
Communication Purpose	(YES/NO)	Specify the mode of communication: Discourse/Single words/Writing/drawing/ Gestures/ Pictograms/Manual Signs/Sign language/Communication Board/Simple communication device/ Complex communication device /Communication Software on a device / Phone./E-mail).
Express needs and desires	☐ YES ☐ NO	
Ask for help	☐ YES ☐ NO	

Show likes and preferences	☐ YES ☐ NO	
Express opinions	☐ YES ☐ NO	
Exchange information	☐ YES ☐ NO	
Discuss ailments	☐ YES ☐ NO	
Express feelings	☐ YES ☐ NO	
Talk to family and friends	☐ YES ☐ NO	
Storytelling	☐ YES ☐ NO	
Talk to people around them	☐ YES ☐ NO	
Have new relationships	☐ YES ☐ NO	

1. Do you think it would be appropriate to work specifically on some aspect of communication with this person?
   ☐ YES ☐ NO

2. Have the disability support services ever worked with the user using Augmentative and Alternative Communication (AAC) devices?
   ☐ YES ☐ NO

   If YES, at what point of their life and what type of communication device has the person used?

3. Do you think that the disability support services are adapted from the point of view of cognitive accessibility for the user? (Information, location, etc.)?
   ☐ YES ☐ NO

   If NO, what aspects could improve?

4. Do you think that the ACC training that is offered in this disability support service is adequate taking into account the person's profile?
   ☐ YES ☐ NO

   If NO, what topics related to ACC training would you suggest?
